# Development and Evaluation of a Computer-Based, Self-Management Tool for People Recently Diagnosed with Type 2 Diabetes

**DOI:** 10.1155/2016/3192673

**Published:** 2016-06-30

**Authors:** Alison O. Booth, Carole Lowis, Steven J. Hunter, Moira Dean, Chris R. Cardwell, Michelle C. McKinley

**Affiliations:** ^1^Institute for Physical Activity and Nutrition (IPAN), School of Exercise and Nutrition Sciences, Deakin University, Geelong, VIC, Australia; ^2^Food and Health Communications, North Yorkshire YO62 6BH, UK; ^3^Regional Centre for Endocrinology and Diabetes, Belfast Health and Social Care Trust, Royal Victoria Hospital, Belfast BT12 6BA, UK; ^4^School of Biological Sciences, Queen's University Belfast, Belfast BT9 5AG, UK; ^5^Centre for Public Health, School of Medicine, Dentistry & Biomedical Science, Queen's University Belfast, Belfast BT12 6BJ, UK

## Abstract

*Aim*. The purpose of this study was to develop and evaluate a computer-based, dietary, and physical activity self-management program for people recently diagnosed with type 2 diabetes.* Methods*. The computer-based program was developed in conjunction with the target group and evaluated in a 12-week randomised controlled trial (RCT). Participants were randomised to the intervention (computer-program) or control group (usual care). Primary outcomes were diabetes knowledge and goal setting (ADKnowl questionnaire, Diabetes Obstacles Questionnaire (DOQ)) measured at baseline and week 12. User feedback on the program was obtained via a questionnaire and focus groups.* Results*. Seventy participants completed the 12-week RCT (32 intervention, 38 control, mean age 59 (SD) years). After completion there was a significant between-group difference in the “knowledge and beliefs scale” of the DOQ. Two-thirds of the intervention group rated the program as either good or very good, 92% would recommend the program to others, and 96% agreed that the information within the program was clear and easy to understand.* Conclusions*. The computer-program resulted in a small but statistically significant improvement in diet-related knowledge and user satisfaction was high. With some further development, this computer-based educational tool may be a useful adjunct to diabetes self-management. This trial is registered with clinicaltrials.gov NCT number NCT00877851.

## 1. Introduction

In 2012, 21 million people in the USA [1] and 2.9 million people in the UK [2] had been diagnosed with diabetes, of whom 90–95% have type 2 diabetes. These rates are expected to increase by over 60% by 2025 [2, 3]. Diabetes is associated with an increased risk of cardiovascular disease and its risk factors including, hypertension, dyslipidemia (high blood cholesterol and triglycerides), and insulin resistance [4], all of which can be attenuated by lifestyle change [5]. Therefore, it is not surprising that diet and exercise are the cornerstones of the management of type 2 diabetes but these self-management aspects can be challenging for patients [6]. With 95% of type 2 diabetes management requiring self-care [7], continuous education is essential.

The UK National Service Framework for diabetes [8] states that “structured and ongoing education, and access to monitoring equipment are vital parts of diabetes care which empower people to effectively selfmanage [*sic*] their condition.” National guidance recommends that structured education should be an integral part of care planning; however its provision, particularly provision of tailored education, may be limited by access to trained educators and competing demands on healthcare staff. The UK Healthcare Commission (2007) reported that only 11% of people with type 2 diabetes had participated in any form of structured education, with attendance varying between 1% and 53% [9]. Opportunities exist to examine innovative approaches for delivery of education to people with diabetes, especially if such approaches can form a useful adjunct to the care offered by health care teams. According to Corben and Rosen (2005) [10], individuals with chronic diseases, including diabetes, are open to innovative health education methods and such individuals have stated that they would like information in as many formats and as early as possible after diagnosis. Computer-based tools represent one such approach that may help to support the diabetes education provided by health professionals. There is some evidence that the use of computer-based education can improve knowledge, motivation, and self-care behaviour in people with diabetes [11–15]. Modest improvements in blood glucose control are also evident [16]. In terms of priority areas for education, in a study of 245 people attending a diabetes clinic at a general hospital in the UK, the topic that respondents overwhelmingly wanted more information on was diet, followed by long-term complications and living a healthy lifestyle [17].

The aim of this study was to evaluate the effectiveness of a computer-based, dietary, and physical activity self-management program for people recently diagnosed with type 2 diabetes on knowledge, attitudes, skills (particularly goal setting), and behaviour.

## 2. Methods

### 2.1. Development of the Program

**Figure 1:**
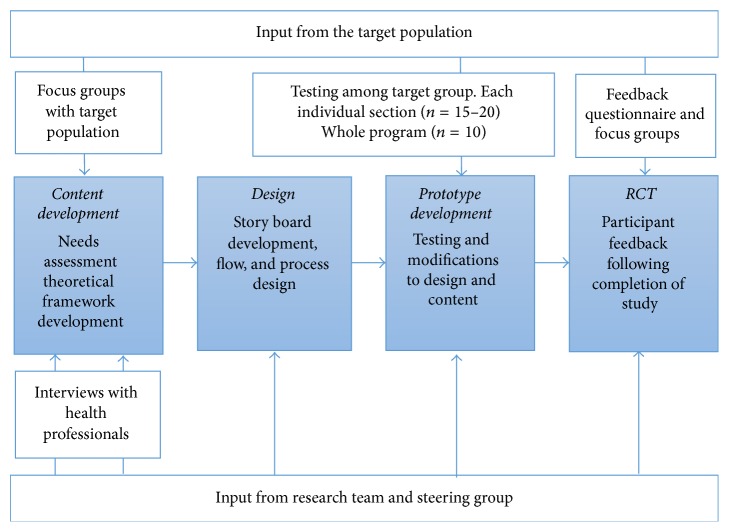
Development of the LWD program.

The program was developed based on existing theoretical frameworks and input from health professionals and the target population (Figure 1) (findings published elsewhere [6]). A qualitative study (structured interviews with health professionals and focus groups with patients) was conducted to gain insight into the issues that needed to be addressed by the program [6]. Overall, the LWD program focused on improving knowledge and addressing misconceptions and encourages self-monitoring of dietary intake and physical activity with goal setting used as a means of facilitating behaviour change. The central theoretical tenet for the development of the program was Bandura's self-efficacy theory [18]. Self-efficacy is recognised as one of the strongest predictors of health behaviour changes and is a component of the majority of psychological theories about behaviour change. A lack of knowledge has been identified as a factor limiting diabetes self-management [19] and as a barrier to behaviour change among people with type 2 diabetes [20]. Goal setting has shown promise for assisting with dietary and physical activity change [21, 22] and has been shown to be an effective behaviour change strategy among people with diabetes.

Five stand-alone sections of the program were developed: (1) A “food diary” allowed the user to record their food and drink intake, receive feedback on the balance of their diet, set goals, and save their data so they could revisit and review their goals. (2) A tailored “activity analyser” assessed current level of moderate and vigorous activity and provided feedback according to the users' stage of behaviour change [23] and encouraged setting goals to help them meet physical activity recommendations. (3) The “fast facts” section contained information presented in a dictionary style and covered a range of topics related to type 2 diabetes, diet, and physical activity. (4) The “quick quiz” consisted of a series of quizzes each composing of 6 multiple choice questions, with immediate feedback, and was designed to allow individuals to assess their levels of knowledge on a range of topics and to address common misconceptions. (5) The “diabetes stories” section included short video clips of three people with type 2 diabetes talking about their experiences of managing their diabetes.

The development process is outlined in Figure 1. A prototype version of each section of the LWD program was developed and each section was then tested (with exception of the diabetes stories section which was not tested owing to time restrictions) by 15–20 participants who had diabetes and the research team. The individual sections were modified based on the feedback from testing and a complete version of the LWD program was compiled. This complete version was tested with 10 patients in order to examine overall ease of use and navigation. Some further minor modifications were made based on feedback from this testing and a full working version was produced for evaluation in the RCT described below.

### 2.2. Evaluation of the Program, a RCT

#### 2.2.1. Recruitment

Ethical approval for the RCT was obtained from the Office of Research Ethics Committees Northern Ireland (ORECNI). Participants were invited to participate from primary care settings in the Belfast area as well as within the Regional Centre for Endocrinology and Diabetes, Royal Group of Hospitals, Belfast. Inclusion criteria were diagnosis of type 2 diabetes within the previous 24 months, access to a computer, and being stable on medication (i.e., no change in medications for the past 2 months). The exclusion criteria were being pregnant or lactating and having a medical condition where changes in diet or physical activity would be contraindicated.

#### 2.2.2. Study Design

The study was registered on ClinicalTrials.gov identifier NCT00877851. After obtaining informed consent, participants were randomised to either the intervention group or the control group. The computer generated randomisation schedule was implemented using consecutively numbered envelopes and was stratified by recruitment site.

The intervention group received the “Life with Diabetes” program on an external hard drive (a USB stick) in order to allow setting, saving, and reviewing goals. Participants received a brief, 10-minute overview of the program and were asked to use the LWD program for 12 weeks. As a minimum level of usage, participants were encouraged to use the two self-monitoring and goal setting aspects of LWD (i.e., the food diary and activity analyser) at least once a week. Participants received a 2-3-minute phone call from the researcher (AB) at around week 4 and week 8 to answer any questions they had about the program and to encourage continued and regular usage. Participants were asked to keep a log detailing their usage of the program (date, length of time, sections used, and additional comments) throughout the intervention period. The control group received a list of useful web site addresses including Diabetes UK and were advised to continue with their usual care, consulting their health care team as they would usually do, for 12 weeks. The control group received the LWD program after completion of the 12-week study.

#### 2.2.3. Primary and Secondary Endpoints

Primary endpoint was between-group differences in diabetes knowledge and setting and achieving goals. Secondary endpoint was between-group differences in dietary intake, physical activity levels, anthropometry, markers of cardiovascular risk (including blood pressure, lipids (HDL, LDL, triglycerides)), blood glucose control (HbA1c, fasting blood glucose), overall self-efficacy, and barriers to the management of diabetes.

#### 2.2.4. Measurements

A baseline questionnaire recorded demographics, frequency and location of computer use, details of diabetes including month/year of diagnosis, current medications, whether participants had attended a group education session after diagnosis, where they received dietary information from, whether they had met with a dietitian, and if they had ever been advised on food intake and/or exercise by a health care provider.

#### 2.2.5. Anthropometric, Clinical, and Biochemical Measurements

The following were assessed at baseline and completion: weight was measured, without shoes and outdoor clothing and after removal of heavy pocket items such as wallets and keys, on a calibrated scales. Weight was recorded in kilograms to the nearest 0.5 Kg. Height was measured without shoes on a stadiometer and was recorded in centimeters to the nearest 0.1 cm. BMI was calculated from weight and height [weight (Kg)/(height (m))^2^]. Waist and hip circumference were measured over light clothing using an inelastic but flexible tape measure. Waist was measured at the midway point between the iliac crest and lower rib. Hip circumference was measured at the widest point around the gluteal protrusion. Blood pressure was measured twice from the right arm, using an automated Omron sphygmomanometer with the participant sitting quietly for at least five minutes. A 20 mL fasting blood sample was drawn from the antecubital vein and was processed within 2 hours. All samples were stored at −70°C for batch analysis at the end of the study. Dietary intake was assessed by 4-day food diary. Physical activity was assessed using the International Physical Activity Questionnaire [24].

#### 2.2.6. Other Questionnaires

Knowledge of type 2 diabetes was examined using a shortened version of the validated Audit of Diabetes Knowledge questionnaire [25]. Scores are represented as percentage correct. Self-efficacy was examined using the validated Diabetes Empowerment Scale [26] with three validated subscales (managing the psychosocial aspects of diabetes, assessing dissatisfaction and readiness to change, and setting and achieving diabetes goals); each question is rated on a 5-point scale from strongly agree [5] to strongly disagree [1]. Barriers were assessed using a modified version of the validated Diabetes Obstacles Questionnaire (DOQ) [27]. Eleven additional statements were included that covered barriers and beliefs identified during focus group disucssions with the target populations during the development phase of this project. Each question is rated on a 5-point scale from strongly agree [1] to strongly disagree [5]. Diabetes-specific quality of life was measured using the validated Audit of Diabetes-Dependent Quality of Life [28]. Scoring ranges from −9 (maximum negative impact of diabetes) to +3 (maximum positive impact of diabetes). Generic quality of life was measured using the validated 36-item short-form health survey (SF36) [29]. Scoring ranged from 0 (lowest level of quality of life) to 100 (highest level of quality of life). Depression was evaluated using the validated, brief, 9-item Patient Health Questionnaire (PHQ-9) [30]. Depression Severity was as follows: 0–4 = none; 5–9 = mild depression; 10–14 = moderate depression; 15–19 = moderately severe depression; 20–27 = severe depression.

#### 2.2.7. Evaluation of the “Life with Diabetes” Program

At the end of the study, all participants in the intervention group completed a program evaluation questionnaire and were asked to participate in an optional focus group discussion. The aim of the focus groups was to gain some further in-depth feedback on the program. Focus group discussions were tape recorded and transcribed verbatim.

### 2.3. Statistical Analyses

Statistical analysis was carried out using SPSS for Windows, version 16 (SPSS Inc., Chicago, IL). A *P* value ≤ 0.05 was considered to be statistically significant. Data was examined for normality of distribution and no transformation of the data was necessary. Baseline characteristics between groups were compared using an independent samples *t*-test for continuous variables and a chi-square test for categorical variables where appropriate. Analysis of covariance (ANCOVA) was used to examine the effect of the intervention. In such analyses the 2 groups (intervention or control) were included as independent variables, and the baseline values were included as covariates. Paired samples *t*-tests were used to examine within-group changes in the study outcomes. Response to intervention was examined according to level of usage as a secondary analysis. Wilcoxon signed ranks test assessed movement between physical activity rankings (e.g., from low to moderate or high to moderate activity level). Pearson chi-square assessed differences between groups in change in activity rankings. As an additional analysis, the intervention group was split into “high users” and “low users” according to the LWD log books; those who reported using the program at least 12 times during the 12-week study were classified as “*high/normal users*”; those who reported using the program less than 12 times during the 12-week study and those who did not return a log book were classified as “*low users.*” ANCOVA was used to compare the outcomes of high versus low users.

## 3. Results

### 3.1. Sample

**Figure 2:**
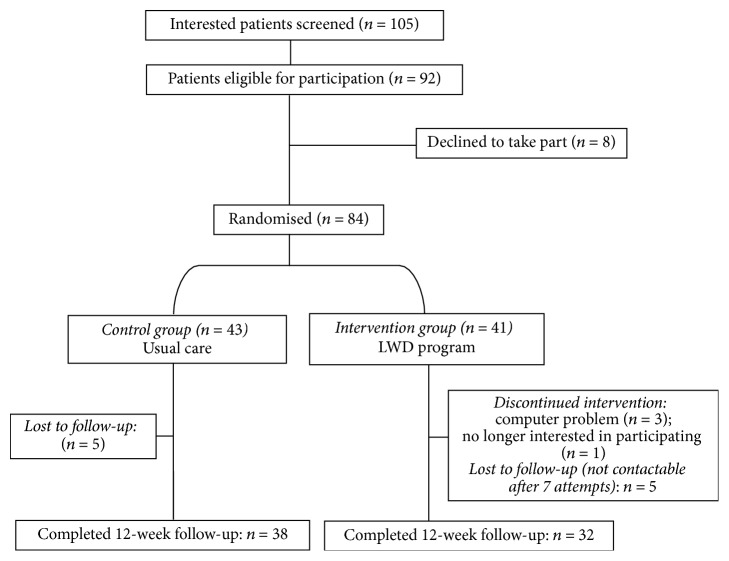
Flow of participants through the RCT.

Figure 2 shows the flow of participants through the RCT. One hundred and eight people expressed interest in the study. Of these, 105 were screened (3 were not contactable after a minimum of 6 attempts), 92 were eligible to participate, eight individuals declined to participate before randomisation took place, and 84 were randomised (41 intervention, 43 control). Seventy participants (84.5%) completed the 12-week study (*n* = 32 out of 41 (78%) intervention group; *n* = 38 out of 43 (88%) control group). Baseline characteristics of all participants are presented in Table 1.

### 3.2. Primary Outcomes

There was no between-group difference in overall ADKnowl score from baseline to postintervention (Table 2) and no statistically significant improvement in the “diet and food scale” of the ADKnowl questionnaire in the intervention group compared to the control group (*P* = 0.09). There was also no significant between-group difference in the “setting and achieving goals” subscale of the DES5 on completion of the intervention.

### 3.3. Secondary Outcomes

There was a significant between-group difference in the “knowledge and beliefs scale” of the DOQ, with an improvement in the “knowledge and beliefs scale” in the intervention group, compared to the control group (mean (SD) 3.5 (1.0) versus 3.3 (0.6), resp.). There were no significant differences between groups after adjusting for baseline variables, for diabetes-specific quality of life (ADDQoL), depression (PHQ-9), or any of the anthropometric measurements or blood parameters shown in Table 3 after the intervention.

Overall there was no significant between-group change in dietary intake during the intervention (Table 4). There was a trend towards a between-group difference in carbohydrate intake, with intake decreasing in the intervention group and increasing in the control group (mean difference *P* = 0.058).

There was a trend for more people in the intervention group to change their category of physical activity in a positive way (moving from low to high, low to medium, or medium to high) during the intervention, with 33% of participants in the control group moving positively between categories compared to 22% of the control group. Figures for changing category of physical activity in a negative way (moving from high level of activity to medium level of activity, high to low, or medium to low) were 5% for intervention group and 16% for the control group. Overall, however, there was no significant difference between groups in change in physical activity over time.

### 3.4. “High” or “Low” Users Compared with Control Group

For the ADKnowl treatment subscale, there was a significant difference between the high users (62% correct) and control group (42% correct) at week 12 (*P* = 0.032 after adjusting for baseline values) but no difference between the low users (53%) and control (42%), *P* = 0.347. For the DOQ, the advice and support scale, there was also a difference between the high users (4.1 (SE) (0.5)) and control group (3.7 (0.6)) (*P* < 0.01 after adjusting for baseline values), but not between the low users (3.5 (0.7)) and control group (3.7 (0.6); *P* = 0.98). Both the high and low user groups scored higher on the “knowledge and beliefs scale” of the DOQ compared to the control group over the 12 weeks (3.7 (0.8), 3.6 (1.1), and 3.3 (0.6), *P* < 0.05 after adjusting for baseline values). There were no other statistically significant differences between the high users, low users, and the control group for any of the other parameters.

## 4. Participant Evaluation of the “Life with Diabetes” Program

### 4.1. Evaluation Questionnaire

All participants in the intervention group completed the evaluation questionnaire. A third of participants rated the overall program as “very good,” half of the participants rated the overall program as “good,” and 16% rated it as “acceptable.” Ninety-two percent would recommend the program to others. Ninety-six percent of participants agreed that the information within the program was clear and easy to understand. Eighty-eight percent agreed that the program was easy to navigate. Some additional free text comments were included in the free text element of the questionnaire: “very encouraging,” “thought it was great,” “very helpful,” and “motivating.”

### 4.2. Focus Groups

Two focus groups were held, each with four participants. All focus group participants had positive views of the program. Many found the program “very motivating,” “excellent,” “very useful,” and “very helpful” and stated that they felt it would keep them “focused and determined” and that it was “easy to use.” They agreed that the format of the program (memory stick) was “handy” because of “the sheer ease of access.” Participants also highlighted that while they find visiting a dietitian to be helpful, they appreciated being able to expand on those sessions by referring back to the program as they wished: “you're aware it's always with you when your dietitian's not” and “sitting for half an hour with the dietitian doesn't really help you much as having that food diary.”

In addition to being the most highly rated section, the food diary was the most frequently discussed section. The food diary appeared to alert participants to healthier eating and areas of their diet they could improve. For example, “you think you're eating very healthily and all of a sudden you realize you're not,” “it made me see that I was actually missing out on some things,” and “it made me very alert to healthier eating.” There were some indications of attempts at making dietary changes. For example, “you think next time I'll have like beans or peas with this,” “trying to get a better balanced diet,” or “tying to eat fewer biscuits.” However, some admitted that they stuck to their traditional diets out of habit or a lack of motivation: “I'll eat the vegetables. Butter is my downfall” and “my diet isn't varied – hasn't changed for many years so I didn't change … I take the blame for that.”

After being asked what they found most enjoyable about the program, the quick quiz section was mentioned by most participants: “it was good to have something interactive” and “I did find the quiz very, very good.” They would often challenge themselves to improve on their scores: “I got quite competitive until I got good scores.” In addition to being enjoyable, they also “found it helpful” and “very informative.”

The fast facts section was also heavily praised: “I was quite taken that it virtually covered everything,” “they were excellent, certainly that was the section that I really got the most out of,” “I really found that very informative,” “I learnt quite a lot from it,” and “the explanation of terms was very useful.”

While the diabetes stories section was rated as the least helpful of the five sections with a score of just above five out of ten, participants expressed in the focus groups a solid appreciation of this section saying they found it “very helpful” and “reassuring.” A typical quote was “the bit where real people, local people, actually talked you know about having diabetes and how they coped with it. I find that very reassuring” and “when you're diabetic everybody has an opinion especially people who have never had it … it's nicer to hear from people from you know that have had it with the experience of having it.”

Finally, while some participants did find the physical activity section helpful (e.g., “it focused my mind on how I could be more active” and “I will go back to that section and see if I can improve on it”), many did not (e.g., “after a while I just sorta stopped going into it,” “you take 30 minutes of exercise a day and all this and you know you're not doing it and there's no use going back into the activity and … doing that again”). However, some admitted that these may be an excuse for not exercising: “not that I take exercise very much [laughs]. It's probably another reason for avoidance.”

## 5. Discussion

Diabetes education delivered by a team of educators, with some degree of reinforcement of that education at intervals, may provide the best opportunity for improvements in patient outcomes [31]. However, the quality and quantity of self-care education given to each individual are highly dependent on the skills and resources of the healthcare professional delivering that education [32]. In the UK, healthcare providers are increasingly pressurised by government driven targets; hence access to nursing or dietetic time for diabetes education may be limited [33]. Innovative, complementary methods of supporting diabetes education are therefore much sought after. Computer-based technology holds promise as an innovative means of supporting the work of health professionals; it can be used to reinforce and extend the education received in the healthcare setting and is readily accessible therefore facilitating opportunities for use on a regular and frequent basis to enhance knowledge and self-monitor key health-related behaviours.

The evaluation of this newly developed tool to aid self-management of diet and physical activity in people with type 2 diabetes indicated that it was highly valued by users and, alongside this, its short-term use was found to significantly improve barriers related to “knowledge and beliefs” about diabetes. There was also a trend towards a statistically significant improvement in overall health status as measured by SF-36 instrument. A secondary analysis of “high” users compared to the control group demonstrated small but significant increases in the “knowledge and beliefs scale” and the “advice and support scale” of the DOQ and a trend towards significance for ADKnowl “diet and food score.” Although there was no significant change between the intervention and control group in self-efficacy, depression, diabetes-specific quality of life, body weight, or metabolic markers, there were indications of positive changes in dietary intake and physical activity between groups, albeit these were not statistically significant. The results, therefore, indicated that this tool did help patients to improve their knowledge, which is one of the key barriers to dietary self-management among people with type 2 diabetes [34–37]. Previous research has indicated that people with type 2 diabetes particularly want information on diet [17] and while enhanced knowledge will not necessarily always translate directly into a change in behaviour, it represents one positive step towards empowering people to make better choices [38–40]. It has been reported that knowledge-behaviour correlations are increased among individuals with increased self-efficacy and decreased among those with decreased self-efficacy [41]. However, the improvement in knowledge in this study was not accompanied by changes in self-efficacy, perhaps owing to the time-scale of the study or the level of usage of the program as discussed below.

The LWD program was highly rated by participants in feedback obtained from both questionnaires and focus group discussions. This is reassuring given the level of involvement the target group had in terms of the development of the program itself and the extensive usability testing that was undertaken. Including members of the target group in the development process is a robust form of program development and has been shown to improve participation and program success [42, 43]. The main attribute that participants appreciated most was having the LWD program on-hand so they could access it at their convenience. There is evidence that home-based instruction is a preferred-choice to group/classes in diabetes self-management [44]. Home-based instruction allows the individual to schedule educational activities around their existing lifestyle, in their own time, and in the comfort of their own home. In addition to the convenience of home-based instruction, this approach may help with retention and application of information as patients report that information received in face-to-face education sessions, while valuable, is difficult to recall at a later time [6]. Having an education tool on-hand, such as the one delivered here, can help to fill this “gap” in diabetes self-management and could be helpful and reassuring for patients, particularly when check-ups or follow-up appointments are frequently 6 months apart. It was also apparent in the focus group discussions that the participants were enthusiastic towards each individual component of the program, often stating that they found them motivating, reassuring, and helpful and that it kept them focused. Some suggested that they did learn new concepts and did attempt to make changes to their diet. Study completion rates were good, with only 16% being lost to follow-up; however only 40% of the intervention group appeared to use the program on at least a weekly basis. Future research on this program should assess ways to enhance compliance and regular use of the program as this may result in additional benefits. For example, embedding the program within current care packages alongside regular contact with a health professional, whether it be by email, letter, or phone call, may be of benefit. Additional changes might include enhancement of the physical activity component of the tool to focus on self-monitoring and goal setting behaviour which may help to increase engagement with this part of LWD. In general, this interactive program would be well suited to delivery via an “app” platform which would increase ease of access thus enhancing opportunities for engagement and regular use.

With regard to dietary intake, based on initial focus group discussions [6], the LWD program focused predominantly on helping the user to understand how to achieve a balanced diet. The program did not focus on calorie restriction and weight loss and, indeed, although some favourable changes in dietary intake were observed, there was no change in body weight following the intervention. Recent guidelines for management of type 2 diabetes [45] emphasise the importance of weight management as the primary goal for people with type 2 diabetes. Based on the findings of the trial and the increased emphasis placed on weight loss in clinical guidelines, it is clear that the issue of weight management needs to be enhanced within this educational tool.

A challenge with all such tools is likely to be the digital divide. A lack of desire to engage with technology was one of the main factors that influenced recruitment to the study. However, on a positive note, most of the people who took part in this study had never used a computer before and yet still managed to navigate their way through the program with ease which was reassuring as ease of use was a key priority when the tool was being designed. Furthermore, the average age diagnosis for type 2 diabetes is decreasing and computer literacy is increasing [46, 47]. Since inception of this project, significant advances have been made in the use of mobile applications for health management [48]. Although this tool was originally designed to be delivered via personal computer, the new technologies available open up increased opportunities to modify the tool for use on a number of different platforms thereby increasing accessibility and potentially enhancing reach and effectiveness. Conversion to an app format would require a similar process of user involvement as for development of the original program in order to ensure usability is appropriate for the target group who will have specific usability requirements compared to other segments of the population [48].

The study did have some limitations. This was a trial of effectiveness and under these “real-life” circumstances compliance was not optimal; based on usage logs, only approximately half of the intervention group participants used the tool on at least a weekly basis as was recommended at the study outset. The “high user” group may represent a more highly motivated population and hence may not be as generalisable to the general population. However, despite this, some positive changes in knowledge-related parameters perceived health status, diet, and activity were observed and the trial has highlighted the need to focus attention on strategies that could be used to enhance usage, such as exploring alternative modes of delivery, such as app technology, and how this tool could be incorporated into existing diabetes care packages, as discussed above. With regard to the user feedback, only a quarter of the intervention group participants opted to take part in the focus group discussion at the end of the study. Feedback from these sessions was positive; however, it is possible that only the more motivated or interested participants opted to take part and therefore these discussions may not be representative of everyone who used the program. It was encouraging, however, that the positive attitude towards the program displayed in the focus group discussions was, for the most part, consistent with the findings from the evaluation questionnaire that was completed by* all* participants who used the program.

## 6. Conclusion

This short-term evaluation indicated that this newly developed computer-program, focusing on diet and physical activity self-management, was well received by participants and its usage resulted in small but statistically significant improvements in diet-related knowledge, as well as nonsignificant improvements in perceived health status, dietary intake, and physical activity. Based on these findings, further development of this tool is warranted. Consideration needs to be given to how the delivery platform can be widened to maximise impact, how this tool could be incorporated into existing diabetes care packages, and what level of health professional support is required to encourage usage and help maximise effectiveness of the tool. This tool may be a viable adjunct to diabetes self-management and could help to fill an important gap in patient care.

## 7. Implications or Relevance for Diabetes Educators

Health professionals are charged with delivering a large volume of information to patients, often within a limited time slot, and human resource issues can also mean there is limited capacity for patient follow-up. Self-management tools such as this can be a useful adjunct to HCP patient education and support and can help fill important gaps in the care pathway, such as the period between diagnosis and receiving structured education, or extended time periods between patient follow-up appointments. For HCPs to have confidence in the effectiveness of any such tool, however, they should be developed in close consultation with the end users and with HCPs and should undergo rigorous usability testing, as was undertaken during this study, before wider dissemination.

## Figures and Tables

**Table 1:** Baseline characteristics of participants randomly assigned to intervention and control group.

	Control (*n* = 43)	Intervention (*n* = 41)
Age (years) (mean (SD))	60 (10.7)	58 (9.1)
Female gender, % (*n*)	37 (16)	46 (19)
Married or living with partner, % (*n*)	79 (34)	76 (31)
Employment status, % (*n*)	—	—
Employed full time	33 (14)	32 (13)
Employed part time	14 (6)	12 (5)
Retired	40 (17)	34 (14)
Education % (*n*)	—	—
Secondary level	33 (14)	49 (20)
College	16 (7)	7 (3)
University	44 (19)	39 (16)
Ever been to a group education session, yes (%)	26 (11)	51 (21)
Visited a dietitian since diagnosis, yes (%)	65 (28)	73 (30)
Taking medication for diabetes, yes (%)	42 (18)	49 (20)
Taking insulin for diabetes, yes (%)	9 (4)	5 (2)
Weight (Kg) (mean (SD))	90.4 (15.1)	91.6 (15.3)
BMI (Kg/m^2^) (mean (SD))	31.7 (4.9)	32.0 (5.4)
Waist circumference (cm) (mean (SD))	109.8 (11.5)	110.1 (10.7)
Hip circumference (cm) (mean (SD))	115.6 (10.2)	115.0 (11.2)
Waist to hip ratio ((waist cm)/hip (cm)) (mean (SD))	0.95 (0.07)	0.96 (0.06)
Systolic blood pressure (mmHg) (mean (SD))	136 (15.0)	136 (16.0)
Diastolic blood pressure (mmHg) (mean (SD))	82 (9.0)	86 (8.9)

**Table 2:** Primary and secondary outcomes: pre- and postintervention questionnaire data according to randomisation.

	Control		Intervention		Mean difference (CI)	*P* ^∧^
	Baseline *n* = 38	Week 12 *n* = 38	Baseline *n* = 32	Week 12 *n* = 32		
*Primary outcomes*						
ADKnowl: overall score^a^	60 (14.3)	64 (14.2)	62 (13.8)	67 (10.4)	1.7 (−1.7, 5.1)	0.37
ADKnowl: diet and food (item-set 10, 11, 12)^a^	59 (16.0)	60 (18.4)	62 (17.4)	67 (15.8)^*∗*^	5.1 (−0.9, 11.1)	0.09
ADKnowl: effects of physical activity (item-set 8, 9)^a^	40 (26.7)	42 (27.3)	41 (26.2)	47 (24.5)	5.2 (−2.9, 13.4)	0.31
ADKnowl: reducing the risk of complications (item-set 1, 15, 16, 17)^a^	88 (13.1)	89 (12.6)	89 (12.2)	89 (10.4)	−0.7 (−5, 3.6)	0.76
DES5: setting and achieving goals (items 5–14)^a^	3.8 (0.5)	3.8 (0.6)	3.6 (0.8)	3.8 (0.5)	0 (−.29, .29)	0.99

*Secondary outcomes*						
Diabetes obstacles questionnaire (knowledge and beliefs scale)^b^	3.4 (0.5)	3.3 (0.6)	3.1 (0.6)	3.5 (1.0)^*∗*^	0.4 (0.1, 0.7)	**0.01**
ADDQoL: average weighted impact score^c^	−0.9 (1.0)	−0.6 (0.8)	−1.2 (1.3)	−1.0 (1.3)	−0.3 (−0.7, 0.2)	0.28
PHQ-9: overall score^d^	6.2 (5.9)	5.3 (4.7)	8.3 (7.9)	8.0 (8.1)	3.4 (−0.2, 6.7)	0.15
SF36 health survey: overall score	60 (23.8)	69 (18.0)	63 (27.9)	63 (30.4)	−4.8 (−10.4, 0.7)	0.09
SF36 health survey: mental health	69 (20.5)	70 (17.0)	65 (24.1)	61 (28.7)	−4.7 (−10.8, 1.4)	0.90
SF36 health survey: physical health	55 (25.2)	61 (18.4)	61 (26.8)	60 (29.5)	−3.9 (−9.0, 1.0)	0.12
SF36 health survey subscale: physical function	62 (31.1)	68 (26.9)	73 (29.7)	73 (31.9)	−0.6 (−7.7, 6.4)	0.86
SF36 health survey subscale: role limitations due to physical health	54 (44.4)	72 (38.6)	66 (44.2)	61 (47.1)	−14.9 (−29.3, −0.6)	**0.04**
SF36 health survey subscale: general health	53 (23.4)	58 (18.1)	55 (26.3)	55 (27)	−1.7 (−8.3, 4.8)	0.59
SF36 health survey subscale: vitality	48 (22.9)	52 (21.1)	51 (24.5)	51 (24.6)	−1.6 (−8.5, 5.3)	0.65
SF36 health survey subscale: social functioning	73 (28)	87 (20.1)	74 (31.7)	73 (34.7)	−12.2 (−20.8, −3.6)	**0.006**
SF36 health survey subscale: role limitations due to mental health	64 (41.8)	81 (36.5)	60 (46.7)	62 (48.2)	−11.7 (−28.5, 5.1)	0.17

^a^Percent of items correct (SD).

^b^Diabetes Empowerment Scale, scored from 1 to 5; a lower score indicates greater goal setting ability/confidence.

^c^Scored from −9 (maximum negative impact) to +9 (maximum positive impact).

^d^Patient Health Questionnaire (depression) score; a higher score indicates higher severity of depression.

^∧^ *P* value, between-group comparison at week 12, adjusted for baseline (ANCOVA).

^*∗*^Within group change, *P* < 0.05 (paired samples *t*-test).

**Table 3:** Secondary outcomes: anthropometric and blood parameters before and after intervention^1^ according to randomisation.

	Control		Intervention		*P* ^*∗*^
	Week 0 *n* = 38	Week 12 *n* = 38	Week 0 *n* = 32	Week 12 *n* = 32	
Weight (Kg)	89.4 (14.9)	89.4 (14.7)	89.4 (13.7)	89.8 (13.8)	0.47
BMI (Kg/m^2^)	31.3 (4.6)	31.3 (4.5)	31.3 (5.1)	31.4 (5.0)	0.81
Waist circumference (cm)	109.0 (11.7)	108.5 (11.6)	108.4 (10.1)	107.5 (9.8)	0.56
Hip circumference (cm)	114.9 (8.9)	114.3 (8.8)	114.2 (10.5)	114.4 (11.2)	0.47
Waist: hip	0.95 (0.07)	0.95 (0.07)	0.95 (0.06)	0.92 (0.16)	0.16
Systolic blood pressure (mm/Hg)	137 (15.2)	137 (15.1)	137 (14.9)	138 (14.2)	0.89
Diastolic blood pressure (mm/Hg)	82 (8.5)	83 (8.6)	85 (7.4)	85 (9.2)	0.83
Total cholesterol (mmol/L)	4.3 (1.2)	4.2 (1.0)	4.4 (1.07)	4.3 (0.96)	0.52
HDL cholesterol (mmol/L)	1.2 (0.27)	1.2 (0.33)	1.2 (0.32)	1.2 (0.30)	0.75
Calculated LDL cholesterol (mmol/L)	2.3 (0.97)	2.1 (0.91)	2.4 (0.93)	2.2 (0.77)	0.66
Triglycerides (mmol/L)	1.9 (0.70)	1.9 (1.04)	1.7 (0.75)	1.9 (0.94)	0.36
HbA1c (DCCT%)	6.4 (1.1)	6.5 (1.1)	6.4 (0.9)	6.4 (0.8)	0.94
Fasting glucose (mmol/L)	8.2 (2.5)	8.1 (2.3)	7.7 (1.8)	8.3 (1.9)	0.12
Insulin (mU/L)	12.8 (7.9)	16.2 (14.9)	15.4 (13.8)	19.8 (25.1)	0.43

^1^Data given as mean (standard deviation).

^*∗*^Between groups at week 12 after controlling for baseline (one-way ANCOVA).

**Table 4:** Pre- and postintervention nutrient intake according to randomisation^1^.

	Control		Intervention		Mean difference (CI)	*P* value^*∗*^
	Baseline	Week 12	Baseline	Week 12		
Energy (Kcal)	1626.8 (494.0)	1697.2 (592.6)	1858.5 (453.6)	1804.7 (429.4)	28.8 (−257, 314)	0.841
Fat (% total energy)	33.3 (4.8)	30.6 (5.8)	31.6 (7.1)	33.0 (6.9)	2.1 (−1.0, 5.3)	0.182
Protein (% energy)	19.0 (3.5)	18.6 (3.0)	17.5 (2.8)	18.6 (3.6)	0.5 (−1.2, 2.2)	0.542
Carbohydrate (% energy)	43.5 (6.8)	47.4 (7.0)	45.3 (6.1)	43.2 (7.6)	−3.5 (−7.2, 0.1)	0.058
Sugars (g)	65.9 (25.7)	75.2 (29.5)	85.1 (31.4)	80.2 (43.6)	9.1 (−14.9, 21.7)	0.712
Sodium (mg)	2548.8 (897.3)	2710.4 (1263.1)	2771.1 (851.1)	2587.1 (868.3)	−259 (−853, 334)	0.384
Fibre (g)	13.7 (5.2)	15.9 (7.3)	13.4 (16.1)	14.8 (4.9)	−1.7 (−4.9, 1.5)	0.285

^1^Data given as mean (standard deviation).

^*∗*^Between groups at week 12 after controlling for baseline (one-way ANCOVA).
